# Colony Expansion of Socially Motile *Myxococcus xanthus* Cells Is Driven by Growth, Motility, and Exopolysaccharide Production

**DOI:** 10.1371/journal.pcbi.1005010

**Published:** 2016-06-30

**Authors:** Pintu Patra, Kimberley Kissoon, Isabel Cornejo, Heidi B. Kaplan, Oleg A. Igoshin

**Affiliations:** 1 Department of Bioengineering, Rice University, Houston, Texas, United States of America; 2 Department of Natural Sciences, Del Mar College, Corpus Christi, Texas, United States of America; 3 Department of Natural Sciences, University of Houston-Downtown, Houston, Texas, United States of America; 4 Department of Microbiology and Molecular Genetics, University of Texas Medical School, Houston, Texas, United States of America; University of Illinois at Urbana-Champaign, UNITED STATES

## Abstract

*Myxococcus xanthus*, a model organism for studies of multicellular behavior in bacteria, moves exclusively on solid surfaces using two distinct but coordinated motility mechanisms. One of these, social (S) motility is powered by the extension and retraction of type IV pili and requires the presence of exopolysaccharides (EPS) produced by neighboring cells. As a result, S motility requires close cell-to-cell proximity and isolated cells do not translocate. Previous studies measuring S motility by observing the colony expansion of cells deposited on agar have shown that the expansion rate increases with initial cell density, but the biophysical mechanisms involved remain largely unknown. To understand the dynamics of S motility-driven colony expansion, we developed a reaction-diffusion model describing the effects of cell density, EPS deposition and nutrient exposure on the expansion rate. Our results show that at steady state the population expands as a traveling wave with a speed determined by the interplay of cell motility and growth, a well-known characteristic of Fisher’s equation. The model explains the density-dependence of the colony expansion by demonstrating the presence of a lag phase–a transient period of very slow expansion with a duration dependent on the initial cell density. We propose that at a low initial density, more time is required for the cells to accumulate enough EPS to activate S-motility resulting in a longer lag period. Furthermore, our model makes the novel prediction that following the lag phase the population expands at a constant rate independent of the cell density. These predictions were confirmed by S motility experiments capturing long-term expansion dynamics.

## Author Summary

Collective motility is a key mechanism bacteria use to self-organize into multicellular structures and to adapt to various environments. An important example of such behavior is social (S) motility in the gram-negative bacterium *Myxococcus xanthus*. S-motile cells are restricted to movement in groups and do not move as individual cells. S-motility is powered by type IV pili (TFP)–multi-subunit filaments, which extrude from the cell poles, adhere to the substrate and retract, pulling the cell forward. TFP retraction or adhesion is suggested to be triggered by extracellular exopolysaccharides (EPS) deposited by cells on the substrate. As individual cells synthesize both pili and EPS, it is unclear why S-motile cells only exhibit group movement. Moreover, the experimentally observed initial cell-density dependence of S-motility remains unexplained. To understand these phenomena, we developed a mathematical model for the colony expansion of S-motile cells. Our model hypothesizes that the EPS level regulates the TFP activity that initiates collective cell movements. With this assumption, the model quantitatively matches the density-dependent expansion rate. Moreover, the model predicts two phases during colony expansion: an initial density-dependent lag phase with a slow expansion rate, followed by a faster expansion phase with a density-independent rate. These model predictions were confirmed by long-term colony expansion experiments.

## Introduction

New interest in the study of microbial collective behaviors has been ignited by recent discoveries that are critical to bacterial pathogenesis and multicellular developmental processes in these single-cell organisms, including quorum sensing [1, 2], phenotypic heterogeneity [3], and biofilm formation [4]. The soil bacterium, *Myxococcus xanthus* is the premiere bacterial model organism for investigations of self-organization and multicellular development [5]. Different *M*. *xanthus* multicellular behaviors emerge depending on their environmental conditions. In nutrient-rich conditions, *M*. *xanthus* cells spread in a coordinated manner forming organized groups [5]. When spreading over prey microbes, *M*. *xanthus* cells self-organize into bands of traveling waves termed ripples [6–8]. When nutrients are scarce, *M*. *xanthus* executes a multicellular developmental program in which roughly 100,000 cells aggregate into a hay stack-shaped fruiting body within which many of the cells sporulate [9, 10].

*M*. *xanthus* cells move exclusively on solid surfaces and this movement is essential for all their multicellular behaviors. *M*. *xanthus* possesses two genetically distinct types of motility: gliding or adventurous (A) motility and twitching or social (S) motility [5, 11, 12]. Single cell movement is facilitated by A-motility, which is most efficient at high agar concentrations. In contrast, group movement is facilitated by S-motility, which is most efficient at low agar concentrations. S-motile cells only move when they are within a cell length of a neighbor [11]. Wild-type cells exhibit these two motility systems simultaneously. This most likely has been a selective advantage enabling *M*. *xanthus* to adapt to a variety of physiological and ecological environments. Both motility systems enable these rod-shaped cells to move along their long axis and periodically reverse direction by switching polarity, i.e. the leading cell pole becomes a lagging pole and vice versa [13–15].

The molecular basis of A and S motility has been studied extensively [5, 11, 12, 16–19]. Recent studies have proposed a ‘focal adhesion complex’ model for A motility in which intracellular motors interact with adhesion complexes on the membrane that are bound to substrate and power movement by pushing against the substrate [19]. S motility has been determined by genetic and behavioral analysis to require interaction between type IV pili (TFP) that powers movement and extracellular matrix polysaccharide (EPS) [16, 20–23]. The lipopolysaccharide (LPS) O-antigen is also required for social motility, yet its contribution is currently unclear [23]. The TFP are filaments 5–7 nm in diameter and 3–10 μm in length composed of PilA monomers encoded by the *pilA* gene. Each step of S movement, involves TFP extension and retraction, which is achieved by polymerization and depolymerization of PilA monomers. Secreted EPS is the anchor and/or trigger for TFP retraction [24]. Consequently, *M*. *xanthus* mutants lacking TFP or EPS fail to display S motility [16, 18, 22, 25].

Although it is clear that TFP and EPS are essential for social motility, many aspects of S motility-driven colony expansion remain unexplained [26]. For example: why, despite of cell reversals, does the colony radius increase [27, 28] and why does the observed colony expansion rate depend on the initial cell density [11, 27, 29]? To explain the S motility-driven colony expansion dynamics of *M*. *xanthus* cells we have developed a mathematical model that accounts for the interaction between TFP and EPS. This model makes two novel predictions that are confirmed experimentally in this report.

## Results

### A reaction-diffusion model for social motility-driven colony expansion

To study social motility-driven colony expansion of *M*. *xanthus* cells we developed a reaction-diffusion model. The major assumptions and ingredients of the model are summarized and justified in this section and the technical details are included in the Methods section.

Experimentally, social motility in *M*. *xanthus* is usually studied by placing a specific number of liquid-grown cells on an agar plate and measuring the increase in the colony diameter over time. Notably, these colony expansion experiments start with over 10^5^ cells and the cell population further increases over time [27, 29]. The colony dimensions (∼10–30 mm) are orders of magnitude larger than the single cell length (4–5 μm). These conditions make it impractical to simulate the expansion using agent-based modeling [30]. Therefore, we focused on continuous approaches formulating the equation for *ρ*(r,t)–cell density at a given location and time. Furthermore, the experimental studies are conducted over a long observation period (∼10–100 hr), which is much longer than the single-cell reversal period (∼5–10 min) [27–29]. Under these conditions, we can approximate the cell movement over a time-scale of multiple reversals as diffusion [31]. An effective diffusion coefficient can be estimated based on the single-cell speed and reversal period (see Methods section) [31].

For S-motile cells, TFP adhesion and/or retraction is stimulated by the presence of EPS [24, 25, 32, 33] and therefore, effective diffusion should increase with increasing EPS. To incorporate this into the model, we used the following expression for the effective diffusion coefficient,

$$D(e)=D_{0}+D_{p}\phi (e).$$ (1)

Here *D*_*0*_ is a S motility-independent diffusion coefficient. For strains lacking A-motility (A^−^S^+^ strains) or wild-type cells under conditions in which A motility is ineffective (low agar concentrations) this term is small and can arise from the mechanical cell-cell repulsion during growth [34, 35]. It can be estimated from the expansion of mutants lacking both A and S motility [27]. In the second term, *D*_*p*_, the maximal diffusion coefficient due to S-motility is multiplied by a dimensionless factor 0≤*ϕ(e)*≤1. This factor is a function of the local EPS concentration (*e*) and can be interpreted as a probability of pilus retraction. We assume that in the absence of EPS retractions fail (*ϕ*(0) = 0) and at high EPS concentrations retractions always succeed *(ϕ(∞)→*1). For our model we have chosen a phenomenological Hill-function of *ϕ(e)*:

$$\phi (e)=\frac{e^{m}}{e_{0}^{m}+e^{m}}$$

where *m* is the Hill coefficient and *e*_*0*_ is half-saturation concentration.

To compute the local EPS concentration, *e*(*r*,*t*) we assume that each cell produces EPS at a constant rate (*α*) with a resulting production flux being a linear function of cell density. We assume that EPS is not diffusible as it consists of large macromolecules that bind to the agar surface and that it degrades/dries with a constant rate (*β*).

If cell diffusion (random motion) was the only factor contributing to cell expansion, we would expect that the colony radius would increase as a square root of the expansion time [36] and correspondingly the expansion rate would gradually decrease. However, this is not experimentally observed [27, 28]; instead, an approximately constant expansion rate is seen. This apparent contradiction can be resolved by our observations that during long-term expansion experiments the cells continue to grow (the *M*. *xanthus* generation time of ~4–5 hr [37–39] is shorter than the typical spreading experiment time-scale). Thus, since cell growth can substantially change the expansion dynamics [31, 39], it must to be accounted for in the model. The growth rate is modeled using the Monod equation

$$g(N)=\frac{g_{max}N}{N_{0}+N}$$ (2)

where *g*_*max*_ is the maximum growth rate, *N* is a local density of growth-limiting nutrients and *N*_0_ is half-saturation coefficient [40]. The nutrients will also diffuse through the agar [41] (the corresponding diffusion coefficient is denoted as *D*_*N*_).

When the assumptions described above are combined together, the following set of three coupled partial differential equations describe S motility-driven colony expansion,

$$\frac{\partial }{\partial t}\rho (r,t)=\underset{cell\;motility}{\underbrace{\frac{1}{r}\frac{\partial }{\partial r}(rD(e)\frac{\partial }{\partial r}\rho )}}+\underset{cell\;growth}{\underbrace{g(N)\rho }}$$ (3)

$$\frac{\partial }{\partial t}N(r,t)=\underset{nutrient\;diffusion}{\underbrace{\frac{1}{r}\frac{\partial }{\partial r}(rD_{N}\frac{\partial }{\partial r}N)}}-\underset{nutrient\;consumption}{\underbrace{g(N)\rho }}$$ (4)

$$\frac{\partial }{\partial t}e(r,t)=\underset{EPS\;production}{\underbrace{\alpha \;\rho }}-\underset{EPS\;drying}{\underbrace{\beta \;e}}$$ (5)

where *D(e)* and *g(N)* are given by Eqs (1) and (2), respectively. To reduce the number of unknown parameters we can without loss of generality set the half-saturation EPS level *e*_*0*_ = 1. This is done by rescaling the EPS level and production rate to *e → e/e*_*0*_ and *α → α/e*_*0*_, respectively. *M*. *xanthus* motility parameters were estimated in a modeling study that showed existence of traveling waves during colony expansion [31]. In this model, a cell density-dependent diffusion rate for cell movement was assumed irrespective of their motility (A or S) type. Whereas our model is based on the experimental observation that the TFP motility (or the diffusion rate) is regulated by the self-produced cellular EPS.

### The colonies expand as a traveling wave with a sharp front

**Fig 1:**
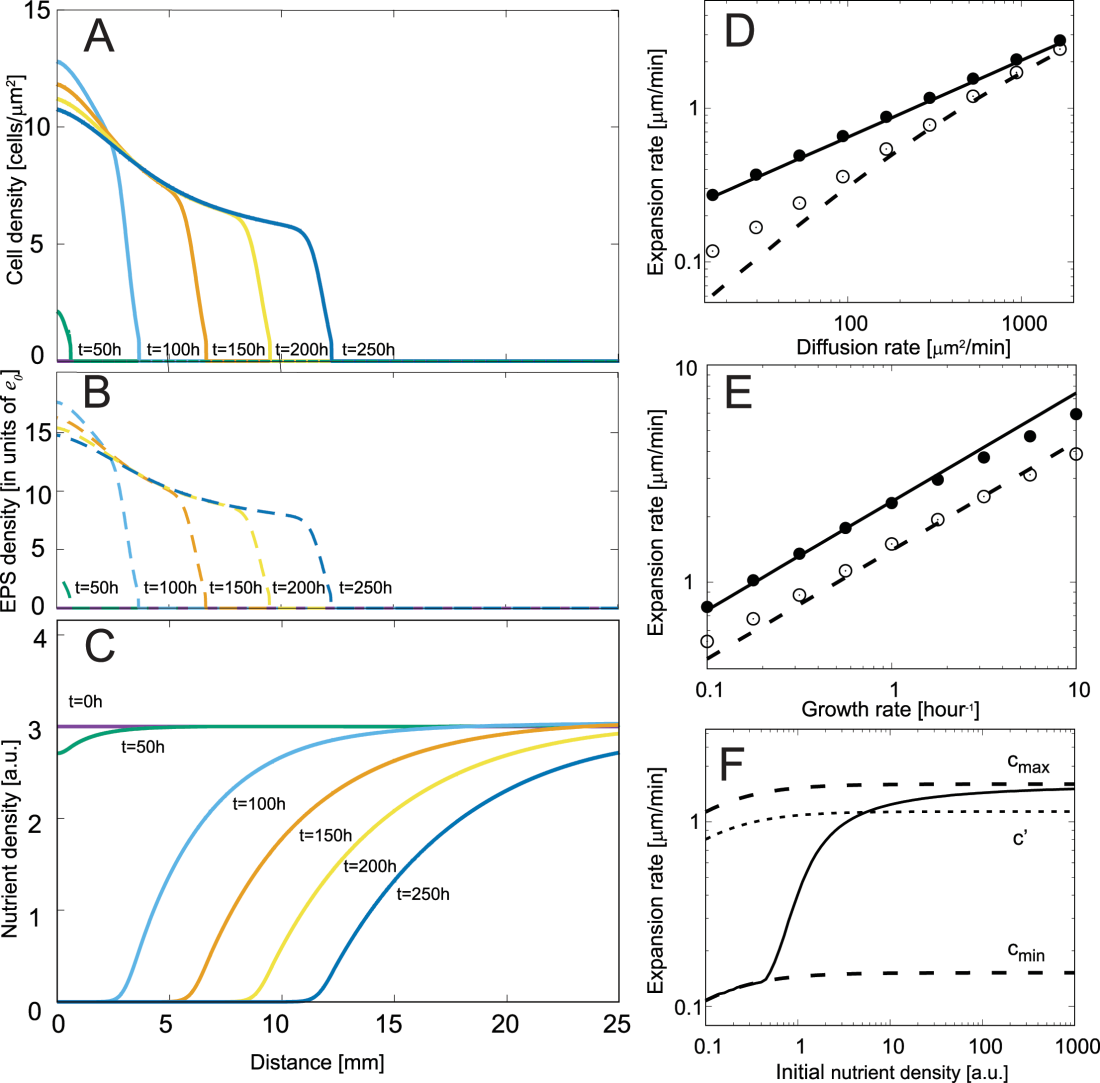
The colony front advances with a constant speed after a transient period. Numerical solution of the model equations (Eqs (3–5)) over a period of 250 hours with the following parameters: *g* = 0.173 *h*^−1^, *D*_0_ = 2 *μm*^2^*min*^−1^, *D*_*p*_ = 220 *μm*^2^*min*^−1^, *D*_*N*_ = 10^4^ *μm*^2^ *min*^−1^, *m* = 4, *α*/*e*_0_ = 19 *h*^−1^, *β* = 16 *h*^−1^. (A) The advance and shape of the colony front (solid lines) between equidistant time points becomes constant at longer times, which indicate that our model equations exhibit a traveling wave solution. The colony front has a sharp edge, which is a consequence of large decrease in diffusion rate below a threshold EPS level, which depends on the population density. (B) The EPS levels (dashed lines) are proportional to population density and display similar travelling wave behavior. (C) The nutrient level decreases with time as the population expands and consumes more nutrients. The nutrient level is measured in arbitrary units ensuring 1:1 nutrient to cell density conversion in Eqs (3–5). (D,E) The long-term colony expansion rate scales similar to Fisher waves (as $\sqrt{D_{p}{\;g}_{max}}$) with the diffusion rate *D*_*p*_ and growth rate *g*_*max*_ for non-diffusing nutrients (*D*_*N*_ = 0) (solid line and closed circles). The circles (closed/open) represent the colony expansion rate computed from numerical solution of model equations and the lines (solid/dashed) represent the expansion rate calculated from a phase-space analytical method (see Methods section). The open circles in 1(D,E) correspond to the expansion rate in the presence of diffusing nutrients and deviate from the square root scaling in *D*_*p*_ and is captured in the expansion rates determined from phase-space analysis (dashed line). (F) The steady state expansion rate increases from a minimum value $\left(c_{min}=2\sqrt{D_{0}g_{max}\frac{N_{in}}{N_{0}+N_{in}}}\right)$ to a maximum value $\left(c_{max}=2\sqrt{(D_{0}+D_{p})g_{max}\frac{N_{in}}{N_{0}+N_{in}}}\right)$ as the initial nutrients level increases. Here *c'* (dotted line) is the expansion rate with the EPS level set to its half saturation level *e*_*0*,_ i.e., the minimum level of EPS at which S-motility becomes active.

We numerically solved the set of equations described above with the appropriate initial and boundary conditions (details are provided in the Methods section). Fig 1A shows the numerical solution of the population density and nutrients at different times. In our simulation, cells enter from the outer edge of the initial colony (at distance *r* = 0 in Fig 1A) into an empty region and grow by consuming the available nutrients (Fig 1A). As the cell density increases, the level of EPS rises, which in turn increases the diffusion rate of the cells and causes the population to spread outward. The population profile shows a sharp increase in density at the colony front (defined as the advancing part of the population profile), which is a consequence of the sharp increase in diffusion rate with an increasing EPS density. The existence of a sharp profile is consistent with the colony patterns observed during social motility [5, 11, 27], where there are no single cells at the colony edge (defined as the low density region leading the advancing colony front). At longer incubation times, the shape of colony front becomes fixed and the colony expansion rate becomes constant, i.e. there is a traveling wave solution. Such properties of the reaction diffusion model with population growth are traditionally observed in Fisher’s equation (also known as Fisher-Kolmogorov equation), which is widely used in theoretical ecology [42]. The equation was first formulated by Fisher to describe the spread of advantageous genes in spatial populations and assumed logistic growth and constant diffusion [43, 44],

$$\frac{\partial }{\partial t}\rho (x,t)=D\frac{\partial^{2}\rho }{\partial \;x^{2}}+g\rho (1-\rho ).$$

This equation admits a traveling wave solution of the form *ρ*(*x*,*t*) = *ρ*(*x* − *c t*), where the wave speed is given by [43, 44]

$$c=2\sqrt{Dg}$$

An extended form of this equation in which the growth rate depends on the nutrient concentration (via Eq (2)), also displays a traveling wave solution [45] with a speed that can be shown (for non-diffusing nutrients, i.e. *D*_*N*_ *= 0*) to be

$$c=2\sqrt{Dg_{max}(\frac{N_{in}}{N_{0}+N_{in}})}$$

[45] (see S1 Text) where *N*_*in*_ is the initial nutrient concentration. In these examples, the expansion rate is determined by the maximum growth rate at the tip of the wave [42] where the population density is low (*ρ*~0) and nutrients are high *N~N*_*in*_.

In contrast to these cases, in our model, the diffusion rate is non-linear and it increases from a low value (*D*_*0*_) at the outer edge to a higher value (*D*_*0*_*+D*_*p*_) in the interior as EPS levels increase from the outer edge to the interior of the colony (Fig 1B). Therefore, the colony interior determines the expansion rate in our model. For Fisher’s equation with non-linear diffusion, an analytical expression of the expansion rate is often not straightforward. However using simple scaling (see S2 Text), we show that the expansion rate is proportional to $\sqrt{D_{p}g_{max}}$ (as shown in Fig 1D and 1E), therefore four-fold changes in effective diffusion or growth leads to two-fold changes in the wave speed. The proportionality coefficient may differ from the value of 2 for the Fisher equation and depends on model parameters *D*_*0*_, *N*_*0*_ and *N*_*in*_. The dependence of the expansion rate on the cell diffusion rate and growth rate is a common feature displayed in colony expansion models for different bacteria [35, 42, 46] including *M*. *xanthus* [31].

By applying traveling wave solutions to our model, we can numerically calculate the exact wave speed using a method of phase-space analysis (see Methods section). The expansion rates determined by the phase-space analysis are in agreement (solid and dashed lines in Fig 1D and 1E) with the expansion rates calculated by measuring the advance of colony front over time with variations in the model parameters (circles in Fig 1D and 1E). For instance, the expansion increases from a minimum value *c*_*min*_ to a maximum *c*_*max*_ as the initial level of nutrients is increased (as shown in Fig 1F). In biologically relevant conditions, colony expansion is observed on nutrient-rich agar and therefore the nutrients are sufficient for the cells to produce EPS at least to its half saturation value to enable S motility-driven movement. Therefore, for S motility-driven colony expansion, the expansion rate will be between *c'* (dotted line in Fig 1F) and *c*_*max*_, which are the expansion rates for constant EPS at half saturation levels and for large nutrients level.

### The model explains experimentally observed density-dependent expansion

Given that the effective diffusion coefficient increases with increasing EPS density, which in turn increases as more cells produce EPS, we hypothesized that these effects could be responsible for the increase in the expansion rate at higher cell densities. To test this hypothesis, we used experimental data from two papers which measured *M*. *xanthus* S motility-driven colony expansion [27, 29]. Briefly, in these experiments *M*. *xanthus* cells at different densities are placed on an agar substrate and as the cells move and divide, the colony expands and its radius increases. The expansion rate is quantified by measuring the difference between the initial and final radius of the colony. The final time corresponded to 8 hr (for expansion rate estimation) in the Kaiser et al. experiments [27] and 24 hr in the Berleman et al. experiments [29]. Using our model equations, we simulated each set of experiments by adjusting our model parameters.

**Fig 2:**
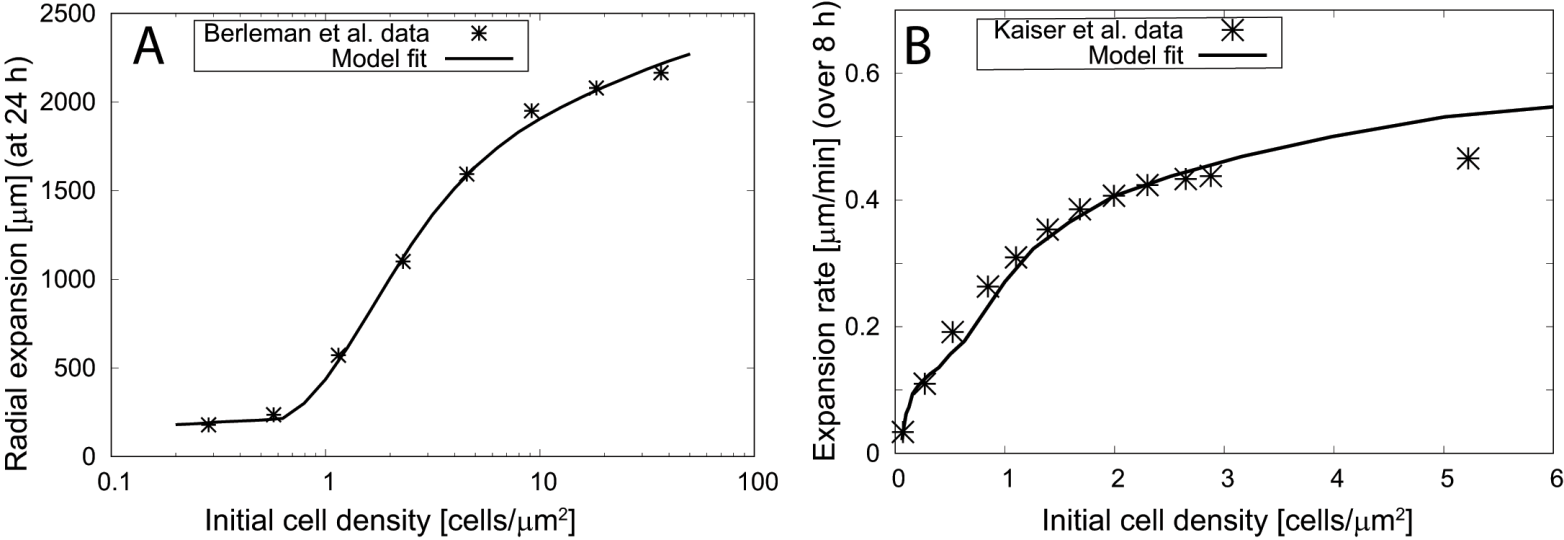
The model fits the density-dependent expansion data from social-motility assay experiments. (A) Colony radius of *M*. *xanthus* strain DZ2 (A^+^S^+^) on 0.5% agar CYE plates after 24 h at different initial cell densities is represented by the stars. The solid line represents the numerical prediction from our model with parameters as in Fig **1**. (B) The expansion rate at different initial cell densities for *M*. *xanthus* A^−^S^+^ strains (an average of three S-motile strains: DK1217, DK1218, DK1219) on 1.5% agar CTT plates at 8 hr is represented by the stars. The solid line represents the numerical prediction from our model in which most of the parameters are the same as in Fig **1**, except *g =* 0.154 *h*^*−1*^,*D*_*p*_ = 16 *μm*^*2*^*min*^*−1*^, *m* = 4, *α/e*^0^ = 150 *h^−1^*.

The results reveal that our model reasonably matches the data from Berleman et al. (Fig 2A) and Kaiser et al. (Fig 2B) which represent a > 100-fold variation in the initial cell density. Notably, the best fit to each data set was achieved using a set of parameters that was identical, except for the maximum diffusion coefficient, *D*_*p*,_ and EPS production rate, *α*. The difference in the effective diffusion coefficients can be easily attributed to the differences in experimental conditions. The Berleman et al. experiments [29] were performed on soft agar (0.5% agar) and led to a high effective diffusion coefficient (*D*_*p*_ = 220 *μm*^*2*^*min*^*-1*^), whereas the Kaiser et al. experiments [27] were performed on harder agar (1.5% agar; *D*_*p*_ = 16 *μm*^*2*^*min*^*-1*^). This is consistent with the fact that S-motile cells perform better on soft agar surfaces. This difference can be achieved with about 3.5-fold differences in the cell speed (see Methods section).

Furthermore, our model predicts differences in EPS-related parameters for the two experiments. To match the data we needed to include an approximately 8-fold difference in the EPS production rate. The same effect can be achieved by changes of the effective EPS drying/degradation rate or the EPS threshold to enable social motility. These results indicate that the production rate or degradation or threshold of EPS could be different for different agar conditions or for the different bacteria strains (A^-^S^+^ [27] and A^+^S^+^ [29]) used in these two experiments. We also noted that the fit was best when the effective cell diffusion *D(e)* sharply changes with the EPS level, i.e. for a high value of Hill’s coefficient (*m≥*4). At lower values of Hill’s coefficient, the model does not fit the experimental data in Fig 2A as it lacks the sharp increase in expansion radius above a threshold initial density (see S3 Text and S1 Fig). This result indicates the existence of a sharp threshold in the EPS level above which TFP are able to attach and/or retract. This sharp threshold is another model prediction that can be tested in the future.

### The model predicts a cell-density dependent lag phase and a constant density-independent steady-state expansion rate

**Fig 3:**
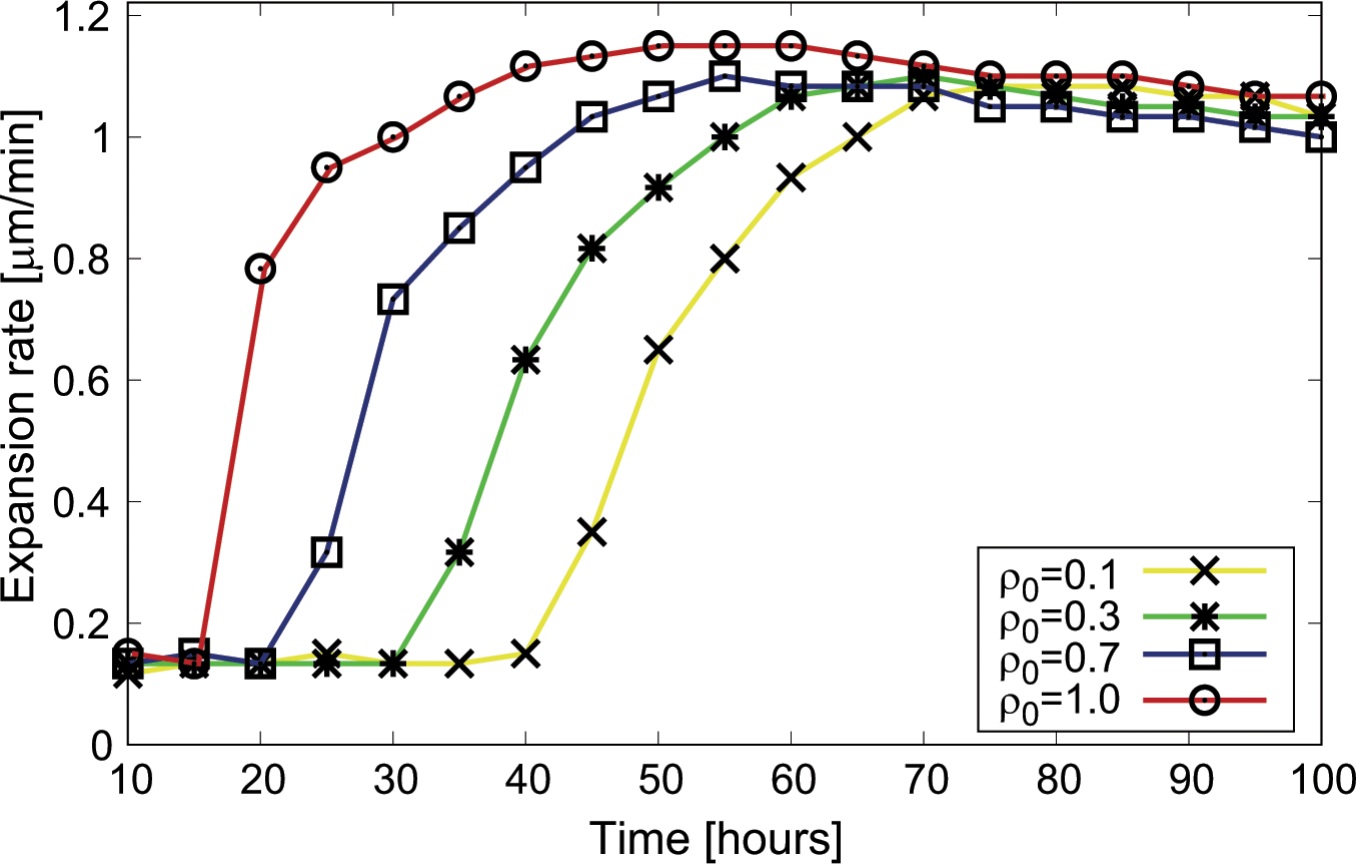
The expansion rate saturates after a transient lag phase that depends on the initial cell density. The model predicts a transient lag phase in the expansion rate with density dependence followed by a constant steady-state expansion rate for all densities. At low density the EPS level is low and slow expansion occurs due to the basal diffusion rate *D*_*0*_. Over time the EPS level increases leading to a steady state phase with a constant expansion rate determined by *D*_*p*_.

To further explore the effects of the different initial cell densities on the colony expansion, we used our model to compute how the expansion rate (defined as the time derivative of the position of the leading edge, where *ρ* is very low ~0.01) depends on time and on the initial cell density for the parameters estimated to match the experimental data used in Fig 2. The results of our simulation (Fig 3) show that the colony expansion rate has a transient slow expansion phase with a duration that depends on the initial cell density, followed by a constant expansion phase at longer incubation times. Our results indicate that a population with a low initial cell density will lag behind higher density populations due to its slower transition to a steady-state expansion rate. This effect is mediated through the production of EPS, which is low for low initial densities. This leads to reduced motility and thereby a slower expansion driven only by the basal diffusion rate *D*_*0*._ The cells start moving with an effective diffusion rate close to *D*_*p*_, only when the EPS density reaches a threshold value. At steady state, the expansion rates for populations that had different initial cell densities are similar because the initial cell density does not affect the cell density of the advancing colony edge.

Previous experiments found the rate of expansion to be different for different initial cell densities and these data suggested that it would remain constant over time [27, 29]. Our model, in contrast, shows that the expansion rate eventually becomes independent of the initial cell density and the density dependence is observed only during a transition period before the constant expansion phase. This contradiction could be due to the fact that previous experiments measured expansion rates for short periods [27], during which some populations were still in their slower expansion phase. Thus, our model, which quantitatively reproduces the previous results, generated a new prediction that could not be confirmed with the previous experimental data. Therefore, new experiments were needed to determine how long the effect of the initial cell density persists during colony expansion.

### A long-term expansion experiment confirms the model predictions

The prediction of a transient density-dependent lag phase followed by a density-independent expansion rate motivated us to conduct systematic long-term colony expansion experiments. Previous *M*. *xanthus* S motility-expansion experiments at different initial cell densities only reported net expansion after 8 hr [27] or 24 hr [29], without reporting any later time-points. To test the prediction we decided to extend these experiments to > 4 days, which according to our model is sufficient to reach the steady-state expansion rate.

The expansion assays were performed on 0.5% agar CTT plates with 0.2% yeast extract and initial cell numbers ranging from about 6 x 10^4^ to 1.2 x 10^7^ cells per initial spot (initial spot radius ~1.7mm). To this end, we inoculated cultures of A^−^S^+^ cells (strain DK1218) and incubated them until an exponentially growing density of ~4 x 10^8^ cells/ml was reached. The cultures were 10-fold concentrated and then diluted to achieve densities ranging from 2 x 10^7^ to 4 x 10^9^ cells/ml. Three μl drops of cells at each density were spotted onto the agar plates and incubated at 32°C. To quantify the colony expansion images of the colonies were collected for at least 96 hr using a stereo microscope and digital camera.

**Fig 4:**
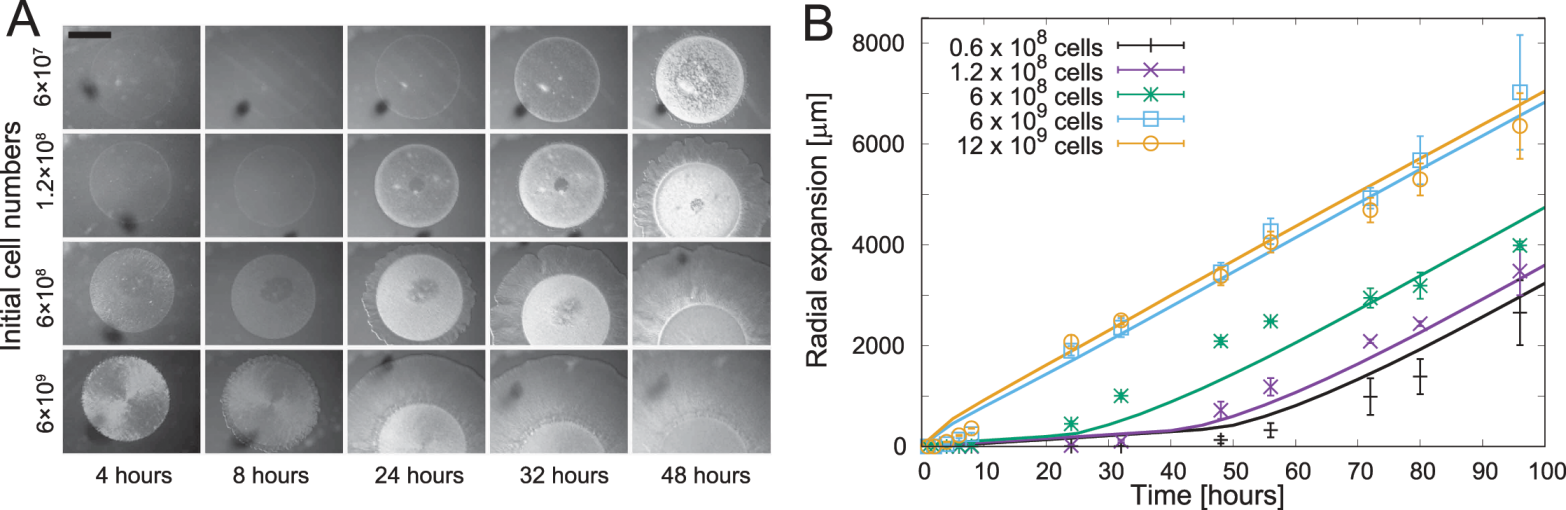
Quantitative analysis of long-term expansion experiments. (A) Cell density-dependent social motility of *M*. *xanthus* strain DK1218 (A^−^S^+^) on 0.5% agar. We observe that the transition from the slow-expansion phase to the constant-expansion phase occurs earlier for cells initiated with high densities as compared to low-density conditions. The bar represents *1700 μm*. (B) The net radial expansion of the colony over time shows that colonies with different initial cell densities (points with standard error) expand with equal rates after an initial transition period, as predicted by the numerical results (lines) of our model.

The increase in colony diameter commenced at different times for different initial cell densities indicating the presence of a density-dependent lag phase. The populations with lower initial cell densities began expansion later than the populations with higher densities, shown in Fig 4A. We performed three replicates of this experiment and quantified the colony expansion by computing the net increase in the colony radius as a function of time for different initial cell densities. We observed that the model fits our data using the same set of parameters as in Fig 2B with the diffusion rates *D*_*p*_ = 200 *μm*^*2*^*min*^*-1*^ due to the use of 0.5% agar. As predicted by the model, the colony expansion during the longer incubation times occurred at a constant rate in our experiments regardless of the initial density (all lines in Fig 4B have equal slopes). Furthermore, we observe that the expansion curve for high initial cell numbers (6 x10^9^ cells & 12 x10^9^ cells in Fig 4B) nearly overlap indicating there is either no lag phase or a very short lag phase at high initial cell densities. These data indicate that cell motility rapidly becomes active due to high EPS production. This scenario directly corresponds to the saturation in the Hill-function (*ϕ(e≫e*_*0*_*)* ~1) at high EPS concentrations and justifies our choice of function *ϕ(e)* to represent TFP activity. Similarly, at low initial cell numbers (0.6 x10^8^ cells & 1.2 x10^8^ in Fig 4B) the differences in the lag phase duration are small, resulting in near overlap of the expansion curves, which suggests that the TFP activity below half saturation (*ϕ(e≪e*_*0*_*)≈(e/e*_*0*_*)*^*m*^) is low until a threshold EPS level is reached. Therefore, our long-term experiments validate the assumptions of our model and confirm its predictions. Moreover, these data reveal the regulation of cell motility by EPS as a mechanistic basis of *M*. *xanthus* social motility.

## Discussion

Reaction-diffusion models have been widely used in many biological systems to study various spatial and temporal patterns [47, 48], including the expansion of microorganisms on surfaces [46, 49, 50]. In this paper, we formulated a deterministic reaction-diffusion model with a key characteristic that cell motility depends on EPS deposition. Our model successfully explains several salient features of S motility-driven colony expansion in *M*. *xanthus*. Specifically, it predicts that *M*. *xanthus* colonies expand as a traveling wave with a sharp front. The speed of expansion scales with $\sqrt{D_{p}g_{max}}$ and its absolute value depends on the EPS half-saturation (*e*_*0*_) and the diffusion rate ratio (*D*_*0*_*/D*_*p*_). Using a calibrated set of parameters the model recapitulates the experimental trends showing density-dependent colony expansion. Our model suggests that, in order to achieve agreement between the modeling results and the experimental trends, a sharp increase in cell diffusion rate at a threshold EPS level is critical. As a consequence the model predicts that populations starting at low initial densities have a lag phase until sufficient EPS accumulates. Our model further predicts that this lag phase ends after longer incubation times and then the population advances at a constant rate irrespective of the initial cell density. To validate these predictions we performed long-term colony expansion experiments with S-motile cells. Our results confirm the presence of a lag phase that depends on the initial density followed by density-independent steady-state expansion.

To explain the experimental data the model assumes that the activity of TFP motility is triggered by the EPS level. To date no definitive evidence proves this relationship. However, experimental studies have reported the loss of social motility in mutants lacking EPS production [25, 29] and the gain of social motility when EPS is complemented externally [29, 33] or when cells are subjected to specific conditions which overcome the EPS requirement (e.g., polystyrene substrate submerged in 1% methylcellulose) [51]. However, it would be useful to show a direct relationship between S motility-driven colony expansion and EPS production. A systematic study could be performed in which EPS mutants are placed on various concentrations of EPS purified from *M*. *xanthus* wild-type cells or mixed with strains producing different amounts of EPS. According to our model, expanding colonies will achieve different expansion rates depending on the EPS level. Using such expansion rate data, the EPS-dependent diffusion rate can be extracted using the relation D(e*) = c^2^*/*2*g* for each known EPS level (e*). Furthermore, examining cell behavior at the colony edge might provide additional insights into this relationship. We expect the cells at the very edge of the colony move less compared to the cells in the interior until the sufficient EPS accumulates. Future quantitative experiments will provide stronger evidence for the role of EPS in the regulation of cell motility.

Although our model is used here to explain social motility-driven expansion in *M*. *xanthus*, the model can also be applied to other bacterial species. For example, a similar density-dependent lag phase is observed during swarming motility in undomesticated strains of *Bacillus subtilis* [52]. Swarming motility is multicellular movement on solid surfaces (soft agar plates in a narrow concentration range: 0.3%-0.5% agar) powered by rotating flagella. *B*. *subtilis* swarming depends on surfactin, which is a surfactant produced by the cells that acts as a lubricant to reduce the surface tension between the cells and substrate, and thereby promotes surface spreading. The surfactant production depends on the local cell-density and appears to regulate colony expansion in a similar fashion as EPS does during social in *M*. *xanthus*. Moreover, the long-term colony expansion rate of *B*. *subtilis* cells lacking surfactant production is shown to be dependent on the amount of the externally provided surfactant levels [52]. Despite these similarities the underlying biophysical mechanism behind the extracellular-component-dependent expansion may be somewhat different. For instance, the surfactin dependency could arise from the fluidic properties of the colony itself. It has been observed that as non-flagellated *B*. *subtilis* colonies expand on hard agar, the colony height (thickness) increases. This transiently increases the osmotic pressure, which eventually decreases as the colony expands. In this case, a non-linear dependence of diffusion rate on the cell density originates from a fluid dynamic model [53].

## Methods

### Model parameters and simulations

We considered most model parameters from the literature [31]. The gliding speed of *Myxococcus xanthus* is reported to be in the range *v*_*g*_ = 4–7 *μm min*^*-1*^ [54]. The reversal period of a single cell varies between 5–10 *min* [13], giving a reversal frequency of *f* = 0.1–0.2 *min*^*-1*^. Single cell movement with periodic reversals can be considered as a velocity-jump process, in which a cell moves along its length at a velocity *v*_*g*_ and reverses direction according to a Poisson process with a constant rate (*1/f*). A diffusion equation can be obtained for such a velocity-jump process and the corresponding diffusion rate can be approximated as *D*_*p*_*≈(v*_*g*_*)*^*2*^*/2f~*80–245 *μm*^*2*^ *min*^*-1*^ [31, 55]. The nutrient diffusion rate in agar media is faster than the cell diffusion rate and is taken to be *D*_*N*_ = 10^4^ *μm*^*2*^*min*^*-1*^[31, 56]. The doubling time is 4 *hr* (DZ2 strain) giving the growth rate *g* = 0.173 *hr*^*-1*^. Other parameters are reported in Fig 1A.

We solve the model equations starting from the edge of the initial spot, which is taken to *r = r*_*0*_ (the initial radius of the colony *~*1300–1700 *μm*) to a boundary (*r_b_* = *r_0_*+30 *mm*). The initial conditions are set as *ρ(r*,*0) = e(r*,*0) = 0* and *N(r*,*0) = N*_*in*_. The boundary conditions are

$${\left(\frac{\partial \rho }{\partial x}\right)}_{r=r_{0}}=c_{0}\rho_{0},\;{\left(\frac{\partial \rho }{\partial x}\right)}_{r=r_{b}}=0,{\left(\frac{\partial N}{\partial x}\right)}_{r=r_{0},r=r_{b}}=0,{\left(\frac{\partial e}{\partial x}\right)}_{r=r_{0},r=r_{b}}=0$$

where *c*_*0*_ = 0.003 *μm*^*-1*^ (10*%* of *v*_*g*_*/D*_*p*_ = 2*f/v*_*g*_, the net flux of cells in the presence of EPS) is the initial flux at which the cells disperse from the edge of the colony and grow by consuming the available nutrients. The initial nutrient profile is set to a uniform value of *N*_*in*_ = 3 *a*.*u*. per μm^2^ and the nutrient half-saturation is chosen to be *N*_*0*_ = 0.1 *a*.*u* per μm^2^ (so that cells stop growing in low nutrient conditions). We numerically solved the partial differential equations (Eqs (3–5)) using the Crank-Nicolson method [57].

### Phase-space analysis for the estimation of traveling wave speed

A phase-space analysis method was used for calculating the steady expansion rate of the traveling waves formed in our model. To simplify the analysis we neglected the radial part of the diffusion term as it decays inversely with expansion distance. In addition we assumed that the EPS concentration quickly reaches its steady state level e* = αρ/β. As a result the model can be reduced to two equations,

$$\frac{\partial \rho }{\partial t}=\frac{\partial }{\partial x}\left(D(\rho )\frac{\partial \rho }{\partial x}\right)+g_{max}\rho \left(\frac{N}{N_{0}+N}\right)$$ (6)

$$\frac{\partial N}{\partial t}=D_{N}\frac{\partial^{2}N}{\partial x^{2}}-g_{max}\;\rho \left(\frac{N}{N_{0}+N}\right)$$ (7)

where $D(\rho )\equiv D(e^{*})=D_{0}+D_{p}\left(\frac{{\left(\alpha \rho /\beta \right)}^{m}}{1+{\left(\alpha \rho /\beta \right)}^{m}}\right)$ is a density-dependent diffusion rate.

In steady state the two model equations display traveling wave solutions. Thus, the following property for *ρ*(*z*) = *ρ*(*x* − *c t*) and *N*(*z*) = *N*(*x* − *c t*) can be considered, where c is the speed of the traveling wave. Using these properties, the equations become

$$-c\frac{d\rho }{dz}=\frac{d}{dz}\left(D(\rho )\frac{d\rho }{dz}\right)+g_{max}\rho \left(\frac{N}{N_{0}+N}\right)$$

$$-c\frac{dN}{dz}=D_{N}\frac{d^{2}N}{dz^{2}}-g_{max}\;\rho \left(\frac{N}{N_{0}+N}\right)$$

Adding the above equations, results in the following

$$-c\frac{d\rho }{dz}-c\frac{dN}{dz}=\frac{d}{dz}\left(D(\rho )\frac{d\rho }{dz}\right)+D_{N}\frac{d^{2}N}{dz^{2}}$$

Integrating once the equations become

$$-c\rho (z)-cN(z)=D(\rho )\frac{d\rho }{dz}+D_{N}\frac{dN}{dz}+IC$$

where IC is an integration constant that can be set by substituting the values *ρ*(*z*) = 0 and *N*(*z*) = *N*_*in*_ at *z* → +∞ (in the unpopulated region). Note that their derivative also vanishes at *z* ± ∞, i.e., *ρ*(±∞) = 0 and *N*(±∞) = 0. As a result IC = −*cN*_*in*_. Therefore, we arrive at the following set of equations

$$N\prime \prime (z)=\frac{\rho g_{max}(\frac{N}{N_{0}+N})-c\;N^{\prime }}{D_{N}}$$ (8)

$$\rho^{\prime }(z)=-\frac{c\;(\rho +N-N_{in})+D_{N}\;N^{\prime }(z)}{D(\rho )}$$ (9)

In the moving *z*-frame, the traveling wave starts from a fixed point (*ρ*(*z*) = *N*_*in*_, *N*(*z*) = 0) at *z* → −∞ and approaches another fixed point (*ρ*(*z*) = 0, *N*(*z*) = *N*_*in*_) at *z* → +∞. To determine the wave speed numerically, we cast the equations above into the following first order autonomous equations,

$$\Omega \prime (z)=\frac{\rho g_{max}(\frac{N}{N_{0}+N})-c\;\Omega }{D_{N}}$$

$$N^{\prime }=\Omega$$

$$\rho^{\prime }(z)=-\frac{c\;(\rho +N-N_{in})+D_{N}\Omega }{D(\rho )}$$

The fixed points of the equations listed above are (*ρ*,*N*,Ω): (0,*N*_*in*_,0) (S2 Fig stable node, closed circle) and (*N*_*in*_,0,0) (S2 Fig saddle node, open circle). The traveling wave solution connects the saddle node or initial state at *z* = −∞ to the stable node or the final state at *z* = +∞ in the phase plane (*ρ*,*N*).

Numerical analysis shows that the solution behavior transitions from oscillatory to negative and then to non-negative values in cell density (*ρ*) as the wave speed *c* increases. As a result, for physically realistic solutions, we need to identify the minimal c value for which the solution becomes non-negative (rather than non-oscillatory). This determines the expansion rate for our model’s equations.

### Cell growth and colony imaging

*M*. *xanthus* strain DK1218 (A^-^S^+^) was grown overnight in CTT broth (1% Difco Casitone, 10 mM Tris-HCl pH 8.0, 8 mM MgSO4 and 1 mM KHPO4 pH 7.6) at 32°C with shaking [58]. When the *M*. *xanthus* culture reached mid-log phase (4x10^8^ cells/ml, 100 Klett units), the cells were harvested in 1.5 ml micro-centrifuge tubes at 13,000 rpm and ten-fold concentrated to Klett 1000 by resuspension in CTT broth. The cells were then diluted in CTT broth to Klett 5, 10, 25, 50, 125, 250, and 500. For microscopy a 3-*μ*L drop of each dilution of *M*. *xanthus* cells was placed onto one 10 cm CTT 0.5% agar plate that also contained 0.2% yeast extract. The plates were incubated at 32°C in an in-house-designed humidity-controlled chamber (a plastic shoe box with a lid in which the bottom was covered by wet paper towels) for more than 96 hrs. Each spot was imaged using an Olympus SZH10 stereo microscope and OptixCam Pinnacle series digital camera with OCView7 software. The distance moved by each colony edge was measured at 2, 4, 6, 8 hrs, and at least twice a day for at least 4 days. Each image was quantified using imaging software GIMP. Specifically, a circle was fitted to the colony in the image to obtain the radius. The expansion distance was computed as a difference between the colony radius at any given time *t* to the initial colony radius. The average and the standard deviation of the expansion distance from three experimental repeats were calculated in an Excel spreadsheet (plotted in Fig 4B). The cell initial density used in the simulation was determined by calculating the number of cells in the drop used for inoculation divided by the average initial colony radius (∼1700 *μm*).

## Supporting Information

S1 Text Phase-space analysis for estimation of traveling wave speed. (PDF)

S2 Text Scaling of traveling wave speed with diffusion and growth rate. (PDF)

S3 Text Model fit at different Hill’s coefficient values. (PDF)

S1 Fig (A) The varition of minimal error with an increase in Hill’s coefficient shows that the model fits reasonaly well with the experimental data for Hill’s coefficients larger than 3–4. (B) For Hill’s coefficient *m* = 4, the model fit matches the observed experimental trend. For Hill’s coefficient *m* = 1, the model fit with the minimum square error does not reproduce the observed non-linearity in the experimental data.
(PDF)

S2 Fig Different phase-space solutions for different wave speeds *c*. (A) An oscillatory stable solution exists when the eigen values at (*ρ* = 0, *N* = *N*_*in*_) have a negative real part and an imaginary part, i.e. when *c* < *c*_*min*_. (B) For *c* > *c*_*min*_ the oscillatory solutions disappear, but a negative solution still exists until *c* is increased leading to a positive solution.
(PDF)
