# Supplementary material for: Colony Expansion of Socially Motile Myxococcus xanthus Cells Is Driven by Growth, Motility, and Exopolysaccharide Production
Source: PLoS Comput Biol. 2016 Jun 30;12(6):e1005010. doi: 10.1371/journal.pcbi.1005010 (PMC4928896; doi:10.1371/journal.pcbi.1005010)
Supplement: S1 Text — (PDF) [file pcbi.1005010.s001.pdf]

Text S1. Phase-space analysis for estimation of traveling wave speed

For the case of non-diffusing nutrients $(D_N = 0)$, some analytical calculations can be made. For this special case, Eq. (5) in the Methods section reduces to

$$N'(z) = \frac{g\rho}{c}\left(\frac{N}{N_0 + N}\right)$$

$$\rho'(z) = -\frac{c\,(\rho\, +\, N -\, N_{in})}{D(\rho)}$$

The shape of the traveling wave is determined by the path connecting the two fixed points in the equations above. The behavior of such connecting paths in the phase plane (ρ, N) can be analyzed by computing eigenvalues around each fixed point: $(0, N_{in})$ and $(N_{in}, 0)$. The eigenvalues λ for the fixed points $(0, N_{in})$ and $(N_{in}, 0)$ are

$(0, N_{in})$: $\lambda = \frac{1}{2D_0}\left[-c \pm \sqrt{c^2 - 4gD_0\frac{N_{in}}{N_0+N_{in}}}\right]$ and

$(N_{in}, 0)$: $\lambda = \frac{1}{2D(N_{in})}\left[-\left(c - g\frac{N_{in}\,D(N_{in})}{c\,N_0}\right) \pm \sqrt{\left(c - g\frac{N_{in}\,D(N_{in})}{c\,N_0}\right)^2 + 4gD(N_{in})\frac{N_{in}}{N_0}}\right]$,

respectively.

It is clear that the solution around the fixed point $(0, N_{in})$ will be oscillatory when $c < 2\sqrt{gD_0\left(\frac{N_{in}}{N_0+N_{in}}\right)}$ and lead to physically unrealistic solutions (negative values for cell density ρ). The solutions lead to a travelling wave with positive values for cell density if the fixed point $(0, N_{in})$ is a stable node; this occurs when $c_{min} \geq 2\sqrt{g\, D_0\left(\frac{N_{in}}{N_0+N_{in}}\right)}$, i.e., a minimum wave speed is required for physically realistic solutions. This condition is a necessary condition for non-oscillatory solutions, but might not always lead to positive solutions in ρ. In our case, the diffusion rate is larger within the wave-front $(\rho > 0)$ than at the leading edge$(\rho \approx 0)$. This leads to three types of solutions in our system, i.e.,

oscillatory, negative, and positive (S2 Fig). The maximum value for the wave speed occurs when the diffusion rate attains maximum value $(= D_0 + D_p)$ rapidly (e.g. when nutrient levels are high or the growth rate is high). The maximum wave speed is given by $c_{max} \geq 2\sqrt{g\ (D_0 + D_p)\ \frac{N_{in}}{N_0+N_{in}}}$. For other parameters, the wave speed lies between these two limits, i.e., $c_{max} > c^* > c_{min}$. It is not possible to compute the exact value of wave speed for our non-linear diffusion rate, but one could obtain wave speed numerically by identifying non-negative solutions.
