# Supplementary material for: Colony Expansion of Socially Motile Myxococcus xanthus Cells Is Driven by Growth, Motility, and Exopolysaccharide Production
Source: PLoS Comput Biol. 2016 Jun 30;12(6):e1005010. doi: 10.1371/journal.pcbi.1005010 (PMC4928896; doi:10.1371/journal.pcbi.1005010)
Supplement: S2 Text — (PDF) [file pcbi.1005010.s002.pdf]

Text S2. Scaling with diffusion and growth rate

We can rescale the Eq. (4) by using $\tau = g\ t$ and $l = \sqrt{g/D_p}\ x$, and obtain

$$\frac{\partial \rho}{\partial \tau} = \frac{\partial}{\partial l}\left(\left(\frac{D_0}{D_p} + \frac{\left({}^{\rho}/_{\rho_0}\right)^m}{1 + \left({}^{\rho}/_{\rho_0}\right)^m}\right)\frac{\partial \rho}{\partial l}\right) + \rho\left(\frac{N}{N_0 + N}\right)$$

$$\frac{\partial N}{\partial \tau} = \frac{D_N}{D_{p_N}}\frac{\partial^2 N}{\partial l^2} - \rho\left(\frac{N}{N_0 + N}\right)$$

The corresponding wave speed will be transformed to,

$$c = \frac{d\bar{x}}{dt} = \sqrt{D_p g}\,\frac{d\,\bar{l}}{d\tau}$$

where, the multiplication factor

$$\frac{d\bar{l}}{d\tau} = f\left(\frac{D_0}{D_p}, \rho_{0,}\frac{D_N}{D_p}, N_{in}\right)$$

depends on the model parameters. For example when $D_0/D_p \ll 1$ and D_N is small, this factor depends on ρ_0 and N_{in} (as shown in the main text Fig 1E).
