# Supplementary material for: Colony Expansion of Socially Motile Myxococcus xanthus Cells Is Driven by Growth, Motility, and Exopolysaccharide Production
Source: PLoS Comput Biol. 2016 Jun 30;12(6):e1005010. doi: 10.1371/journal.pcbi.1005010 (PMC4928896; doi:10.1371/journal.pcbi.1005010)
Supplement: S3 Text — (PDF) [file pcbi.1005010.s003.pdf]

Text S3. Model fit at different Hill's coefficient values

To investigate how the value of Hill's coefficient (m) affects the agreement between the model and the experimental data, we varied three model parameters: Hill's coefficient (m), EPS production (α) and degradation (β) rate, and calculated the error between the model prediction and experimental data in Fig 2A (sum of squares for each data point). The other model parameters were fixed to the value estimated from the literature, as discussed in the Methods Section. For each value of Hill's coefficient, the minimal error (with respect to α and β) was calculated. The minimal error decreases with the increase in Hill's coefficient (S1A Fig) and the best fits are obtained for Hill's coefficient $m \geq 3\text{-}4$. Further increase in Hill coefficient does not significantly decrease the minimal error. As shown in S1B Fig, the model fit with $m=4$ shows the sharp dependence of the expansion on cell density as seen in the experimental data. We chose this value of Hill's coefficient (and corresponding values of α and β) for the rest of the simulations. Note that for lower value of Hill's coefficient (e.g., $m=1$, S1B Fig), the sharpness of the Hill's function is insufficient to make the TFP motility inactive at low initial cell density. Therefore, cells even at low cell density will expand leading to less drastic dependence of the expansion rate on the initial density (S1B Fig). Thus, we conclude that a sharp dependence of motility on the EPS density is required to explain the observed dependence of the expansion rate on the initial cell density.
