# Supplementary figures and images for: Colony Expansion of Socially Motile Myxococcus xanthus Cells Is Driven by Growth, Motility, and Exopolysaccharide Production
Source: PLoS Comput Biol. 2016 Jun 30;12(6):e1005010. doi: 10.1371/journal.pcbi.1005010 (PMC4928896; doi:10.1371/journal.pcbi.1005010)

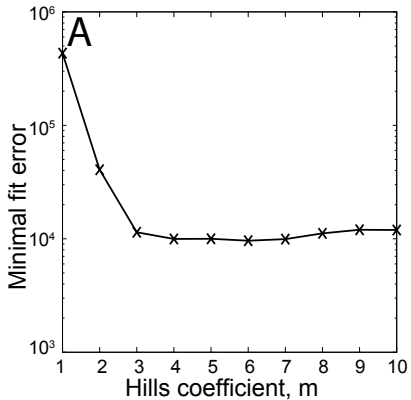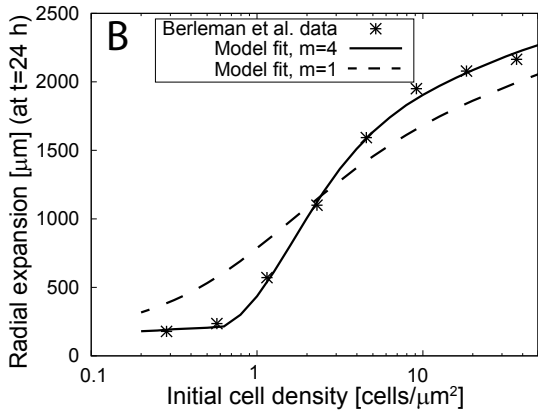

Supplement: S1 Fig — (A) The varition of minimal error with an increase in Hill’s coefficient shows that the model fits reasonaly well with the experimental data for Hill’s coefficients larger than 3–4. (B) For Hill’s coefficient m = 4, the model fit matches the observed experimental trend. For Hill’s coefficient m = 1, the model fit with the minimum square error does not reproduce the observed non-linearity in the experimental data. (PDF) [file pcbi.1005010.s004.pdf]

A

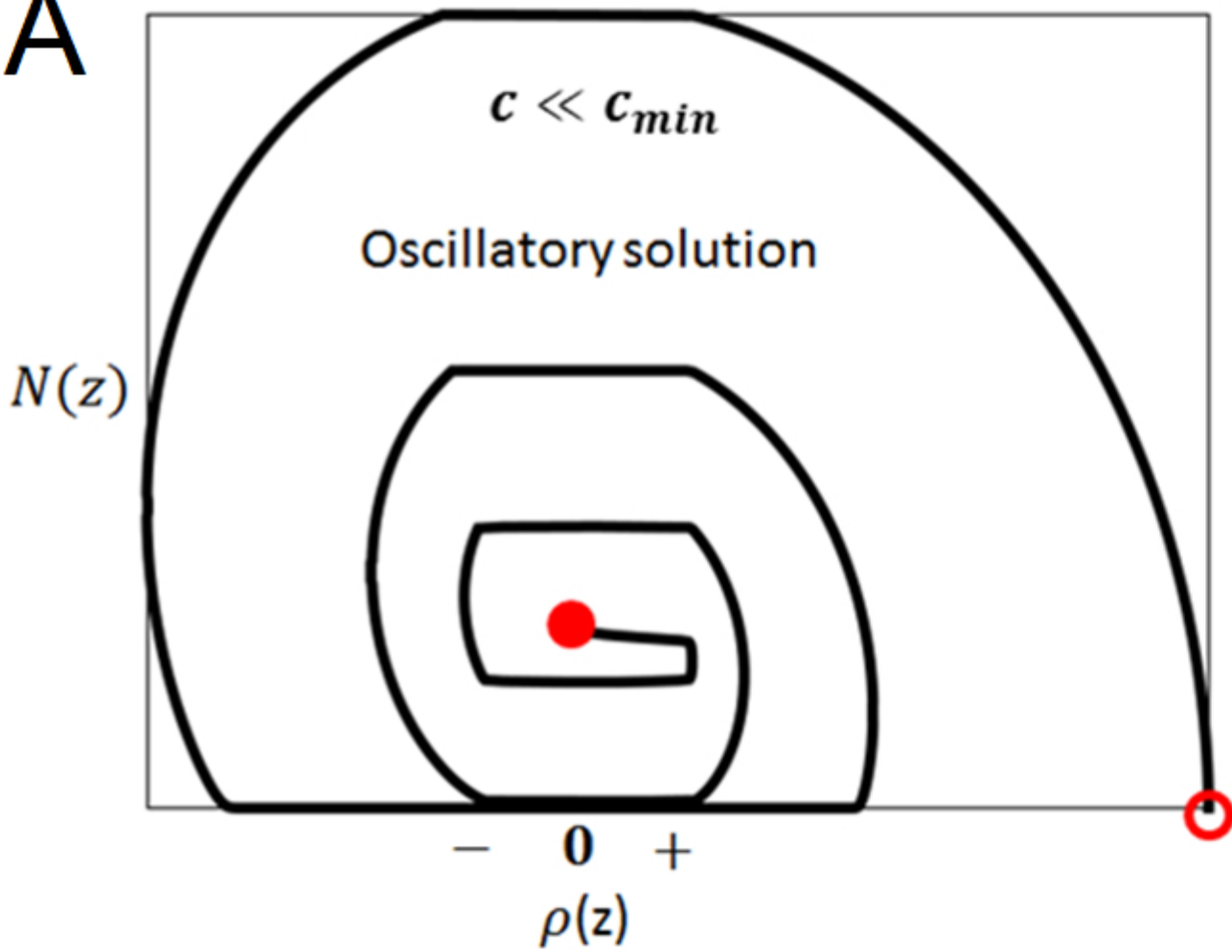

B

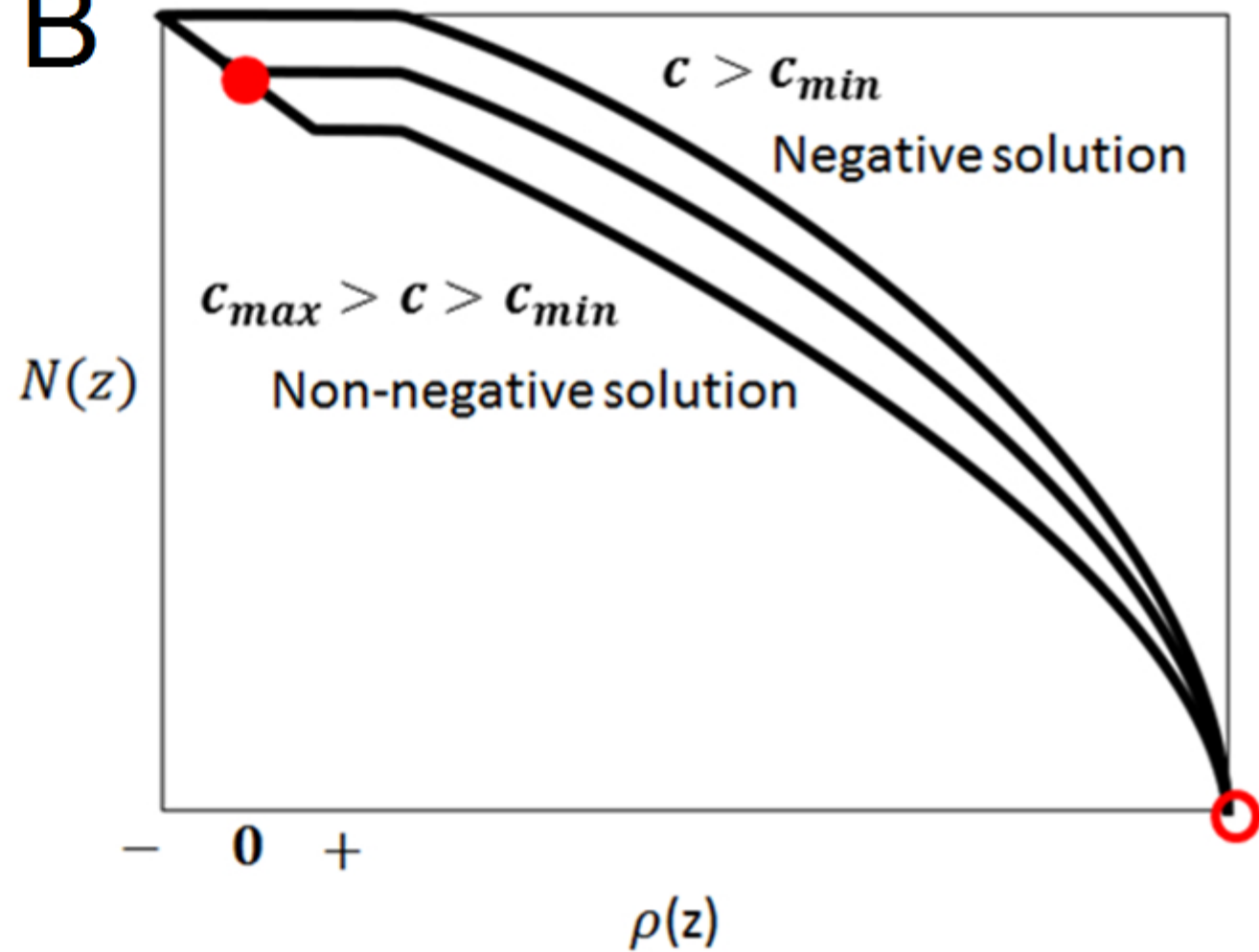

Supplement: S2 Fig — (A) An oscillatory stable solution exists when the eigen values at (ρ = 0, N = Nin) have a negative real part and an imaginary part, i.e. when c < cmin. (B) For c > cmin the oscillatory solutions disappear, but a negative solution still exists until c is increased leading to a positive solution. (PDF) [file pcbi.1005010.s005.pdf]
